# Endocannabinoid-Enhanced “Liking” in Nucleus Accumbens Shell Hedonic Hotspot Requires Endogenous Opioid Signals

**DOI:** 10.1089/can.2018.0021

**Published:** 2018-07-01

**Authors:** Marci R. Mitchell, Kent C. Berridge, Stephen V. Mahler

**Affiliations:** ^1^ Department of Psychology, The University of Michigan, Ann Arbor, Michigan.; ^2^ Department of Neurobiology and Behavior, The University of California, Irvine, Irvine, California.

**Keywords:** anandamide, hedonic hotspot, nucleus accumbens, opioid, pleasure, taste reactivity

## Abstract

**Introduction:** Stimulating either endogenous cannabinoids or opioids within a restricted dorsomedial “hedonic hotspot” in nucleus accumbens (NAc) shell enhances hedonic impact, or “liking” reactions to sweet tastes. In this study, we probed within this hotspot the relationship between endocannabinoid and opioid signals in hedonic enhancement.

**Materials and Methods:** Specifically, we asked whether enhancement of sucrose “liking” by intra-NAc microinjections of the endocannabinoid anandamide requires concurrent endogenous opioid signaling.

**Results:** Co-administration of the opioid antagonist naloxone in the same NAc microinjections with anandamide prevented the endocannabinoid from enhancing orofacial “liking” reactions to sucrose. Since intra-NAc hotspot naloxone injection alone failed to affect hedonics, reversal of anandamide-induced “liking” by opioid blockade reveals an interdependence of opioid and cannabinoid signaling in enhancing taste hedonic impact.

**Conclusions:** These results elaborate our understanding of the mechanisms of hedonic processing of food rewards, and may also carry implications more generally for how opioid and cannabinoid drugs interact to generate natural pleasures, or drug-induced euphoria.

## Introduction

Brain endocannabinoid and opioid signaling systems have overlapping behavioral functions, playing key roles in pain, memory, and reward “liking”. In accordance with this hypothesis, drugs like Δ^9^-tetrahydrocannabinol (THC) and heroin that target these endogenous systems suppress pain, interfere with memory, and have euphoric effects. Cannabinoid and opioid signaling also enhance hedonic “liking” of food rewards, and facilitate incentive motivational “wanting” that, when excessive, can lead to development of compulsive drug seeking in addiction. Given rising use and addiction to opioid and cannabinoid drugs, a better understanding of how these endogenous signaling systems interact in the brain is of significant interest.

The overlapping affective functions of opioids and cannabinoids in nucleus accumbens (NAc) may not be separate, but instead involve synaptic interactions between these signaling systems. For example, cannabinoid receptor type 1 (CBR1) and μ opioid receptors are frequently found in the same cells and afferent axons in NAc shell.^1,2^ Their co-use of Gi/o signaling pathways, as well as their ability to form heterodimers^3^ also point to these signaling systems interacting to modulate behavior.^4–7^ Indeed, there is evidence that cannabinoid and opioid receptor systems interact functionally. For example, cannabinoid antagonists block opioid stimulation of accumbens dopamine release, heroin self-administration, reinstatement of heroin seeking, and food intake.^8–11^ Conversely, opioid antagonists block cannabinoid drug self-administration, the reinforcing effects of THC, and THC-elicited food intake.^12–17^ In addition, cannabinoid and opioid drugs demonstrate cross-sensitization and tolerance, and THC can prime heroin seeking in a self-administration model.^18,19^

We previously reported that NAc microinjections of opioid agonists, as well as the endocannabinoid anandamide, potentiate hedonic “liking” responses to sweet tastes in the taste reactivity paradigm, but only at sites within a 1–1.6 mm^3^ hedonic hotspot located within the rostrodorsal subregion of medial accumbens shell,^20–22^ which is unique within accumbens shell in its anatomical connectivity, its high density of μ opioid receptors, and its dopamine overflow response to opioid agonists.^23–25^ In this study, we asked whether cannabinoid and opioid signaling interact within the hotspot in processing hedonic “liking” of tastes, by testing dependence of anandamide-induced enhancement of “liking” upon local endogenous opioid signaling.

## Methods

Six adult female, and fourteen male Sprague-Dawley rats were pair housed in reverse lights, 12-h light–12-h dark, with *ad libitum* food/water. Experiments were approved by the Institutional Animal Care and Use Committee at the University of Michigan. Females were given bilateral ovariectomy at least 2 weeks before cranial surgery to remove potential influences of ovarian hormones on hedonics.^26,27^ Under ketamine/xylazine (80/7 mg/kg) anesthesia, bilateral 23 gauge, 14 mm guide cannulae were positioned above the NAc shell hedonic hotspot (*n*=10; 3 female), or dorsal control sites at the border of medial prefrontal cortex and rostral accumbens pole (*n*=10; 3 female). An angled track was used to avoid penetration of the lateral ventricles (incisor bar +5 mm; mm relative to Bregma: anterior–posterior [AP]: +3.6, mediolateral [ML]: ±1, dorsoventral [DV]: −5.7). During the same surgery, rats were also implanted with chronic bilateral oral cannulae.^21^ After 7-day recovery, four 60-min habituation sessions were conducted in taste reactivity chambers, followed by four counterbalanced 45-min test sessions when 1 mL of 1% sucrose solution was infused intraorally over 1, 30, and 45 min after the following 0.5 μL intracranial treatments: vehicle (Tocrisolve diluted with artificial cerebrospinal fluid), anandamide (250 ng), naloxone (10 μg), and an anandamide/naloxone cocktail (250 ng/10 μg). Microinjections were delivered through 28 gauge injectors extending 2.5 mm beyond the end of guide cannulae, and tests were conducted 48+h apart.

Immediately after microinjections, a tastant delivery tube (PE-10) was connected to oral cannulae, and rats were placed in the test chamber. At 30 and 45 min post-microinjection, a 1 mL volume of 1% sucrose was infused into the mouth for 1 min, based on previous results indicating hedonic reactions are enhanced by anandamide at those timepoints^21^). Orofacial reactions were video recorded through an angled mirror, allowing clear view of the rats' mouth.

Affective orofacial reactions were coded offline in slow motion by a blinded observer, following established procedures.^21,28,29^ Positive hedonic reactions were considered to be rhythmic midline tongue protrusions, lateral tongue protrusions, and paw licks. Negative aversive reactions were gapes, head shakes, face washes, and forelimb flails,^30,31^ although the last three have also been linked to general activity^32^ as well as aversion. Neutral responses were mouth movements without tongue protrusion. Total hedonic, aversive, and neutral reactions were summed to create composite scores. Following behavioral testing, brains were sectioned, nissl stained, and compared with an atlas to determine microinjection sites.

Repeated measures (drug, time point) and mixed-model analyses of variance (ANOVAs; between subjects variables: sex, cannulae site) were used, with Greenhouse-Geisser correction when sphericity assumptions were violated in Mauchly's test. Post-hoc comparisons employed Tukey's HSD, or corrected *t*-tests. Primary analyses examined total hedonic or aversive reactions to sucrose at both time points (30, 45 min after microinjection), since reactions were similar at both times after intra-NAc microinjections (no main effect of time point on hedonic reactions after NAc drugs; *F*_1,9_=3.76, *p*=0.084; or interaction of drug×time point; *F*_3,27_=0.774, *p*=0.519), as previously reported.^21^ Nor was there a main effect of sex (*F*_1,8_=0.132, *p*=0.726), or interactions of sex with NAc drug (*F*_3,24_=1.664, *p*=0.201), so sexes were combined for subsequent analyses (although this study was not powered to identify subtler sex differences). Significance was set at *p*≤0.05.

## Results

### Anandamide-induced hedonic hotspot depends upon endogenous opioids

**FIG. 1.**
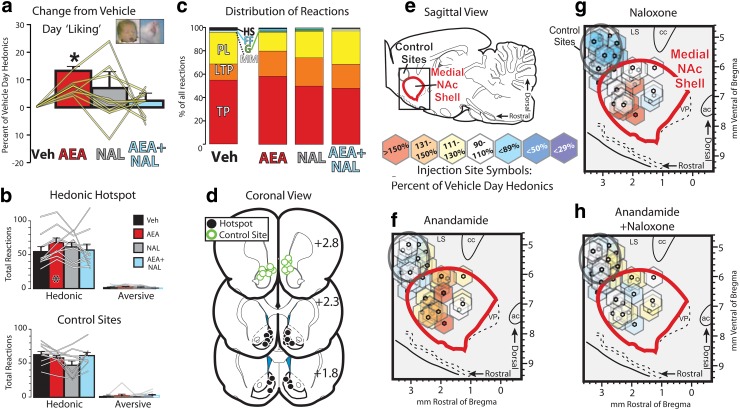
Anandamide-induced hedonics require endogenous opioid signaling in NAc shell hedonic hotspot. **(a)** Percent increase in hedonic reactions to sucrose from vehicle day behavior is shown. Bars indicate group mean and standard errors. Yellow lines represent values for each tested rat with cannulae placed within the medial NAc shell hedonic hotspot. Veh, vehicle; AEA, anandamide, NAL, naloxone; AEA+NAL, cocktail injection of anandamide and naloxone. **(b)** Total hedonic (left) and aversive (right) reactions to sucrose are shown for rats with cannulae in NAc (top) or at rostral control sites (bottom). Group mean/SEMs are represented with bars, and individual rat data are shown with gray lines. **(c)** Distribution of individual scored reactions are shown for each drug condition, with height of each bar representing the mean percentage of all reactions emitted for each drug. Hedonic Reactions: TP, tongue protrusion; LTP, lateral tongue protrusions; PL, paw lick; Neutral Reaction: MM, mouth movement; Aversive Reactions: G, Gape, FF, forelimb flails; HS, head shake. **(d)** Coronal view of cannulae localizations. Cannulae sites of control rats are shown in green, and rats with cannulae in the NAc are shown in black. **(e)** Diagram in sagittal view of the NAc Shell and control sites tested in this study. Hexagons at bottom define the color coding of **(f–h)**, with each color representing the behavioral effect of a drug treatment, relative to that of animals' vehicle day hedonic reactivity. **(f)** Anandamide effects on hedonic reactivity are shown for each rat (placements are represented by hexagons, with size based on putative spread of microinjected drugs in the testing period^21^). Red, orange, and yellow colors represent increases from vehicle day after **(f)** anandamide, **(g)** naloxone, or **(h)** anandamide+naloxone in individual rats. NAc, nucleus accumbens; SEM, standard error of the mean.

Anandamide by itself microinjected into the rostrodorsal NAc shell hedonic hotspot-enhanced hedonic reactions to sucrose, whereas anandamide microinjection in prefrontal cortex or in the rostral pole of NAc, anterior to the hotspot, did not (Drug×cannula site interaction: *F*_1.47,23.52_=5.625, *p*=0.016) (Fig. 1a, b). NAc hotspot anandamide by itself increased the number of hedonic reactions up to 160% of vehicle control levels measured in the same rats on a different day (mean [standard error of the mean] percent of vehicle day=128 (5)%; *t*_9_=7.99, *p*<0.001) (Fig. 1f). By comparison, when naloxone was mixed with anandamide in the same microinjection (*t*_9_=0.87, *p*=0.405), the mixture failed to alter “liking” reactions compared with vehicle day (anandamide+naloxone vs. vehicle: *t*_9_=0.87, *p*=0.405) (Fig. 1a, g, h). Consequently, hedonic reactions were lower when naloxone was mixed with anandamide than when anandamide was delivered alone in the NAc hotspot (anandamide vs. anandamide+naloxone: *t*_9_=2.98, *p*=0.015). However, when naloxone was given alone, hedonic reactions were not altered from vehicle control levels (*t*_9_=1.18, *p*=0.269), indicating that coinjected naloxone reversed anandamide-induced “liking” enhancements, at a dose that did not suppress hedonic impact when microinjected alone. No comparable effects of anandamide or other drugs on hedonics was found after injection into control sites (*F*_1.65,13.17_=3.762, *p*=0.058; a slight decrease in hedonics after control site naloxone drove this ANOVA to approach overall significance), indicating anatomical specificity of effects to the hotspot area.^21^ These results demonstrate that cannabinoid-induced hedonics was blocked by concurrent local opioid receptor blockade in the NAc hedonic hotspot.

No consistent effects of any drugs were found on the low levels of aversive reactivity observed after sucrose (*F*_3,48_=1.15, *p*=0.339), showing specific hedonic enhancement by anandamide restricted here to the positive limb of affect (drug×reaction type interaction for NAc rats: *F*_3,24_=3.0, *p*=0.05). This likely indicates altered hedonic reactivity to anandamide and other drugs, rather than changes in the sensory evaluation of taste, or nonspecific motor effects.

## Discussion

In this study, we demonstrate a previously untested direct link between endocannabinoid and opioid neurotransmission within a dorsomedial NAc shell hedonic hotspot. Microinjections of the endogenous cannabinoid anandamide, or specific μ opioid agonists, when centered in the hotspot region, robustly enhance sucrose “liking”.^20,29^ We show in this study that for anandamide microinjection to enhance hedonics, endogenous signaling at opioid receptors is required, as coadministration of naloxone in the same microinjection prevented anandamide from exerting its hedonic enhancement effects. This is despite the fact that the same dose of the opioid antagonist naloxone, when administered in the absence of anandamide, failed to affect hedonic reactivity—indicating a specific reversal of cannabinoid stimulation effects. This is the first demonstration that “liking” enhancements caused by anandamide stimulation of ventral striatum are interdependent with opioid signals, echoing prior findings that these systems also interact during natural and drug reward seeking, or “wanting”.^4–7^ Anatomical comparison of site effects confirm the dorsomedial NAc shell hedonic hotspot, but not nearby rostral regions of prefrontal cortex or the rostral pole of NAc, in generating anandamide-induced “liking”. This also indicates that, within the NAc shell hotspot, the hedonic-enhancing effects of intra-NAc anandamide depend upon intact signaling at endogenous opioid receptors.

We note that we did not determine whether anandamide effects on “liking” reactions depended upon cannabinoid receptor signaling, and it is possible that anandamide microinjections caused actions at noncannabinoid receptors, such as vanilloid receptors, which anandamide also interacts with.^33^ Since anandamide is typically metabolized rapidly by fatty acid amide hydrolase to metabolites, such as arachidonic acid and ethanolamine,^34^ we also cannot rule out the possibility that hedonic enhancement effects in NAc shell here involve actions of an active metabolite of anandamide, rather than direct actions of the drug itself. The mechanism of anandamide effects in the brain is poorly understood in general, and research on this topic is sorely needed.

Cannabinoids and opioids are tightly-linked receptor systems, and these results show those systems interact in the NAc hotspot to potentiate the hedonic impact of a natural food reward. This hedonic interaction adds to their increasingly studied interactions in pain, addiction, and other pathological processes. Potentially, similar neural interactions could also be involved in generating hedonic components of euphoria from drugs such as cannabis or opiates, which would be of major interest for understanding both the therapeutic and rewarding effects of these classes of drugs.

## Abbreviations Used

ANOVA: analysis of variance
NAc: nucleus accumbens
THC: Δ^9^-tetrahydrocannabinol
